# Inhibitory Effects of Cryptotanshinone and Dihydrotanshinone I on Intracellular Trafficking of Viral Glycoproteins

**DOI:** 10.4014/jmb.2409.09050

**Published:** 2024-10-25

**Authors:** Makoto Muroi, Dong-Sun Lee

**Affiliations:** 1 Antibiotics Laboratory, RIKEN (The Institute of Physical and Chemical Research) 2-1, Hirosawa, Wako-shi, Saitama 351-0198, Japan; 2 Interdisciplinary Graduate Program in Advanced Convergence Technology & Science, Jeju National University, Jeju 63243, Republic of Korea; 3 Faculty of Biotechnology, College of Applied Life Sciences, Jeju National University, SARI, Jeju 63243, Republic of Korea; 4 Bio-Health Materials Core-Facility Center, Jeju National University, Jeju 63243, Republic of Korea; 5 Jeju Microbiome Research Center, Jeju National University, Jeju 63243, Republic of Korea

**Keywords:** Cryptotanshinone, dihydrotanshinone I, antiviral, intracellular glycosylation, trafficking inhibitor

## Abstract

Antiviral agents that target the viral envelope surface glycoproteins can disrupt the interactions between the viral glycoproteins and host cell receptors, thereby preventing viral entry into host cells. However, the mechanisms underlying glycoprotein processing and cellular trafficking have not been fully elucidated. In this study, we aimed to investigate the mechanism of action of cryptotanshinone (CTN) and dihydrotanshinone I (DTN) as inhibitors of viral glycoprotein trafficking, by assessing their inhibitory action on syncytium formation and cytopathic effects. CTN and DTN were isolated and characterized from *Salvia miltiorrhiza*; they effectively inhibited syncytium formation in Newcastle disease virus-infected baby hamster kidney cells. Both compounds inhibited the transport of viral G-proteins to the cell surface, resulting in intracellular accumulation. These results suggest that CTN and DTN are potential glycoprotein trafficking inhibitors that function at the Golgi apparatus. Overall, our results indicate that CTN and DTN suppress intracellular glycosylation by competing as inhibitors of glycosylation trafficking.

## Introduction

Glycoproteins play a major role in various physiological responses in mammalian cells, where they are secreted and transported to the cell membranes via the Golgi complex [1, 2]. The transport of proteins between membrane-bound organelles occurs through a repeated series of budding and fusion of secretory vesicles [3, 4]. Several biochemical and genetic approaches have been used to identify the components of this transport machinery. In particular, the use of in vitro intra-Golgi transport assays has allowed the purification of several cytosolic transport factors [5].

Viral glycoproteins transported to the membranes of infected cells can cause membrane fusion, ultimately leading to syncytia formation. These proteins—including the F protein from measles virus and respiratory syncytial virus, G protein from rabies virus, HA protein from influenza A virus, gp160/gp41 from HIV, and gB protein from herpes viruses—are often found on the surface of enveloped viruses and play a critical role in membrane fusion between infected cells and neighboring uninfected cells [6]. Syncytium formation in Newcastle disease virus (NDV)-infected baby hamster kidney (BHK) cells is caused by the trafficking of viral glycoproteins to the cell surface [7].

Inhibition of viral surface glycoprotein trafficking might be a potential strategy for preventing viral entry and spread. Targeting the glycoprotein trafficking mechanism can interfere with their proper localization to the viral envelope or prevent their transport to the cell surface, thereby reducing viral infectivity. Several approaches have been explored to identify potential inhibitors of glycoprotein trafficking [8]. It has been found that brefeldin A blocks the cell surface expression of viral glycoproteins [9]. Chemical compounds that affect intracellular trafficking are likely to become key tools for unraveling the molecular mechanisms of glycoprotein secretory pathways, subsequently facilitating further investigations of cancer, viral infections, and other degenerative diseases [10, 11]. Viral glycoproteins have served as an excellent model system for studying the biosynthesis, processing, and intracellular translocation of integral membrane proteins [12]. The vesicular stomatitis virus (VSV) model is a particularly useful tool for studying membrane protein translocation, as it shuts off all cellular macromolecular synthesis after viral infection and only one virus-derived glycoprotein, the G protein, is synthesized [13].

Mechanisms of glycoprotein processing and cellular trafficking need to be investigated in detail, as only a few such mechanisms have been partially elucidated. During a previous investigation, while screening for selective inhibitors of viral G-protein transport, we identified and isolated cryptotanshinone (CTN) and dihydrotanshinone I (DTN) from the roots of *Salvia miltiorrhiza* as trafficking inhibitors. Both compounds exhibited various biological activities including antibacterial, antioxidant, and anticancer effects [14-16]. In this study, we examined the inhibitory effects of CTN and DTN on intracellular glycosylation and trafficking of viral proteins in cultured mammalian cells.

## Materials and Methods

### Cells, Viruses, and Reagents

Baby hamster kidney (BHK) cells were cultured in Eagle’s minimum essential medium (MEM; Gibco, USA) supplemented with 10% calf serum (Gibco) at 37°C in a humidified CO_2_ incubator (5% CO_2_, 95% air). The NDV Miyadera strain and VSV New Jersey serotype were obtained from the National Institute of Health (Republic of Korea). BHK cells were separately infected with NDV and VSV. The resulting viral stocks were titrated using a plaque-forming unit (PFU) assay on BHK cell monolayers. VSV stocks were stored at –80°C (Nihon Freezer, Japan). All other chemicals were purchased from Sigma Chemical Co. (USA). CTN and DTN were isolated and characterized from methanol extracts of *Salvia miltiorrhiza*, using various analytical procedures.

### Cell Culture

BHK cells were seeded in 96-well plates, treated with designated two-fold serially diluted concentrations of CTN and DTN, and incubated at 37°C in a CO_2_ incubator. Cell growth was determined by a colorimetric method using WST-1 [2-(4-iodophenyl)-3-(4-nitrophenyl)-5-(2,4-disulfophenyl)-2H-tetrazolium monosodium salt](Wako, Japan), according to the manufacturer’s instructions [17]. Cell growth was determined daily for 3 days and duplicate samples were used for each determination.

### Syncytium Formation and Cytopathic Effects

Syncytium formation (SF) and cytopathic effects (CPE) in the NDV- and VSV-infected BHK cells were observed under a light microscope [15, 16]. Infectious virus production was quantified and expressed as cytopathic units (CPU). The medium fraction of VSV-infected BHK cells in each well was serially diluted to two-folds and added to the BHK cells in 96-well microtiter plates. CPU was expressed as the maximum number of dilutions tolerated to induce CPE.

### Hemagglutination and Hemadsorption

Synthesis of the NDV hemagglutinin-neuraminidase (NDV-HN) glycoprotein was quantified by determining the hemagglutination units (HAU) in whole lysates of NDV-infected cells [17-19]. NDV-HN expressed on the cell surface was quantified by hemadsorption (HAD) as described previously [17-19]. The cell surface expression of the NDV-HN glycoprotein was determined by HAD as follows. Confluent cultures of BHK cells in 6-well plates (Falcon; BD, USA) were infected with 2 ml of NDV at a concentration of 1 HAU/ml and incubated for 14 h at 37°C in a humidified 5% CO_2_ incubator. The medium was removed by aspiration, and then 2 ml of 1% (v/v) chicken red blood cells in chilled saline was added to each well; the plate was kept at 4°C for 30 min with occasional gentle stirring. Unadsorbed red blood cells were removed and the cell layers were rinsed three times with 2 ml of cold saline. The adsorbed red blood cells were swollen in distilled water containing 1% ammonia and quantified by measuring absorption at 550 nm.

### Analysis of Viral Proteins

BHK cells were grown in a 96-well multiplate (Nunclon, Denmark) and infected with VSV at an input multiplicity of approximately 10 PFU/cell. After 2 h of incubation at 37°C, the medium was replaced with a methionine-restricted medium (1.5 μg/ml) with or without CTN and DTN. After 1 h, [^35^S]-methionine (0.37 MBq/ml) was added to the cultures, which were further incubated for 2 h. The labeled cells were chased for 1 h in presence of excess non-labeled methionine (150 μg/ml) and cycloheximide (1 μg/ml). VSV proteins in the cell and medium fractions were analyzed by SDS-polyacrylamide gel electrophoresis (SDS-PAGE) using a 10% acrylamide gel [20].

### Fluorescence Icroscopy

VSV particles were isolated from the culture broth of VSV-infected BHK cells, as described by Kelley *et al*. [21]. Viral G-protein was extracted using phosphate buffer containing Triton X-114 [17, 18]. Briefly, rabbits were immunized with G protein, and IgG was produced from the antiserum. BHK cells were plated on glass coverslips and kept overnight. Sparse cultures were infected with VSV. After incubation for 1 h at 37°C, CTN and DTN were added to the cultures along with cycloheximide (1 μg/ml) and incubated again for 1 h. The cells were fixed overnight in PBS containing 3% paraformaldehyde (w/v) and washed with PBS containing 10 mM glycine. The cells were permeabilized for 5 min in methanol or PBS containing 0.1% Triton X-100, and treated with PBS containing 0.5% bovine serum albumin to rule out non-specific absorption. After exposing the cells to anti-rabbit IgG antibody (1 μg/ml) for 1 h, they were washed with PBS. The cells were then exposed to FITC-conjugated secondary antibody (50 μg/ml) for 1 h and washed again with PBS. Coverslips were mounted on glass slides in 90%glycerol and 100 mM Tris-HCl (pH 7.2). The cells were then photographed using a fluorescence microscope (Zeiss, Germany) at the Bio-Health Materials Core Facility, Jeju National University (Republic of Korea).

## Results

### CTN and DTN Inhibit Syncytium Formation

**Fig. 1:**
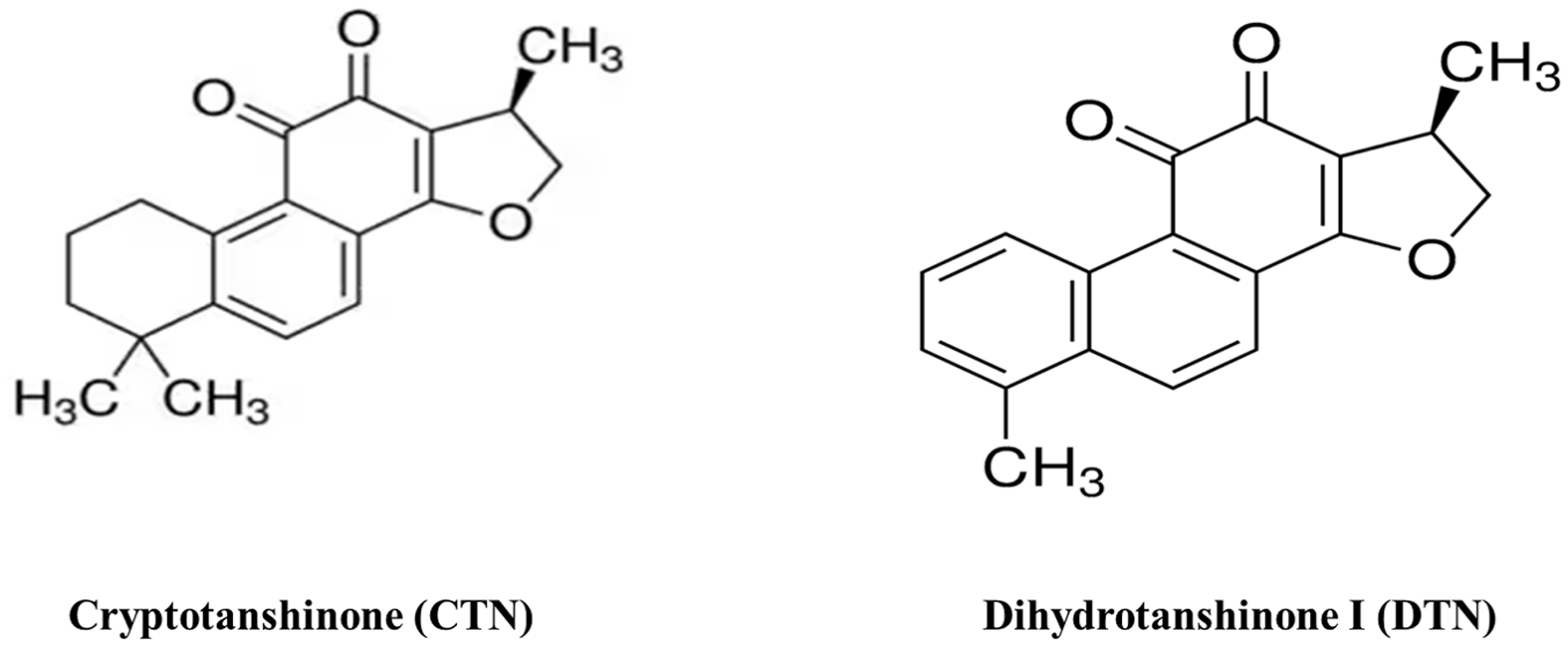
Structures of cryptotanshinone and dihydrotanshinone I.

Confluent monolayer cultures of BHK cells in microtiter plates were infected with NDV or VSV. About 1 h after infection, CTN and DTN (Fig. 1) were added at the specified concentrations, and the incubation was continued for an additional 18 h. We found that CTN and DTN suppressed SF with minimum inhibitory concentrations of 20 and 10 μg/ml, respectively (Table 1). CPE and infectious virus production in VSV-infected cells were strongly inhibited over a similar range of drug concentrations. The concentrations of both compounds at which SF was strongly inhibited, we observed that viral glycoprotein biosynthesis was partially inhibited, as measured by the quantitative analysis of hemagglutination (HAU). The synthesis of both NDV and VSV glycoproteins is dependent on the cellular machinery. Therefore, CTN and DTN inhibited CPE and infectious virus production in VSV-infected cells, although VSV-G was not quantified in this experiment. This is supported by the observed intracellular accumulation of VSV-G in the presence of CTN and DTN (Fig. 1). The synthesis of viral glycoproteins under these experimental conditions reflected the synthesis of viral mRNA, followed by the synthesis and glycosylation of viral proteins. This was because CTN and DTN were added at an early stage of viral growth. The results suggest that CTN (20 μg/ml) and DTN (10 μg/ml) can inhibit SF and infectious virus production without profoundly affecting the synthesis of virus-encoded macromolecules.

### Effect of CTN and DTN on Cell Surface Expression of Viral Glycoproteins

**Fig. 2:**
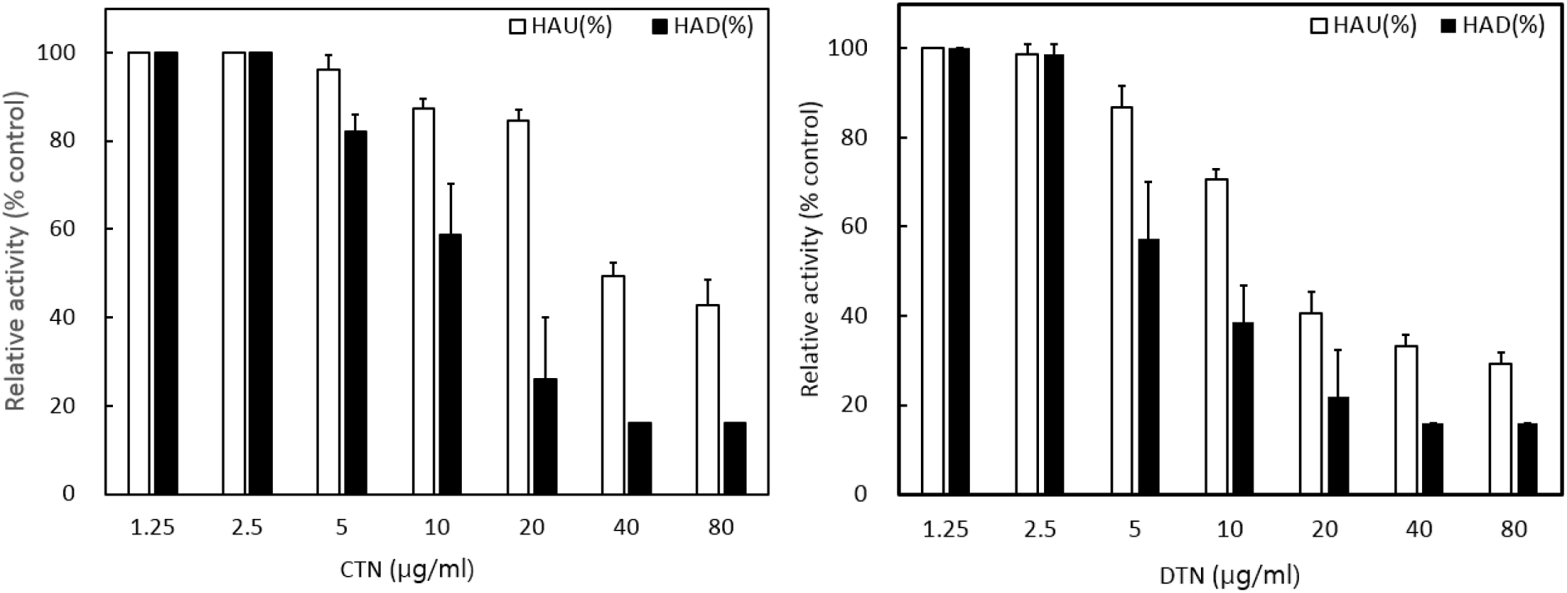
CTN and DTN block cell surface expression of NDV-HN glycoproteins without significantly affecting their synthesis. Monolayer cultures of BHK cells in 6-well or microtiter plates were infected with NDV, and CTN or DTN were added to the cultures at the indicated concentrations, hours after infection. %HAU (□) and %HAD (■) were determined at 14 h after infection. Synthesis of NDV-HN protein was quantified by determining HAU in whole lysates of infected cultures in microtiter plates, and cell surface expression was quantified by measuring the number of chicken red blood cells adsorbed to intact infected cells in 6-well plates. The results are expressed as a percentage (%) of the control value. Abbreviations: BHK, baby hamster kidney; NDV, Newcastle disease virus; CTN, cryptotanshinone; DTN, dihydrotanshinone I; HAU, hemagglutination units; HAD, hemadsorption; NDV-HN, NDV hemagglutinin-neuraminidase.

To determine whether these compounds are potent SF blockers without profound effects on glycoprotein synthesis, the effects of CTN and DTN on the cell surface expression of viral glycoproteins were examined. BHK cells in 6-well plates were infected with NDV and incubated at 37°C for 12 h in the presence of CTN and DTN at the specified concentrations (Fig. 2). Total and cell surface-expressed NDV-HN glycoproteins were quantified based on hemagglutination and hemadsorption activities, respectively. To quantify the total amount of synthesized NDV-HN, whole NDV-infected cultures were disrupted by brief sonication. Chicken red blood cells were added to determine hemagglutination in the lysates. A drastic decrease in HAU in the presence of CTN and DTN was not observed at any of the concentrations up to 20 and 10 μg/ml, respectively (Fig. 2, □ ). However, the binding of extracellularly added chicken erythrocytes to the surface of intact NDV-infected cells, expressed as %HAD, decreased as a function of the concentration of CTN and DTN (Fig. 2, ■ ). This indicated that CTN and DTN blocked the cell surface expression of the NDV-HN glycoprotein in a dose-dependent manner. Taken together, these results suggest that CTN and DTN can block the cell-surface expression of NDV-HN without any profound effect on its synthesis.

**Fig. 3:**
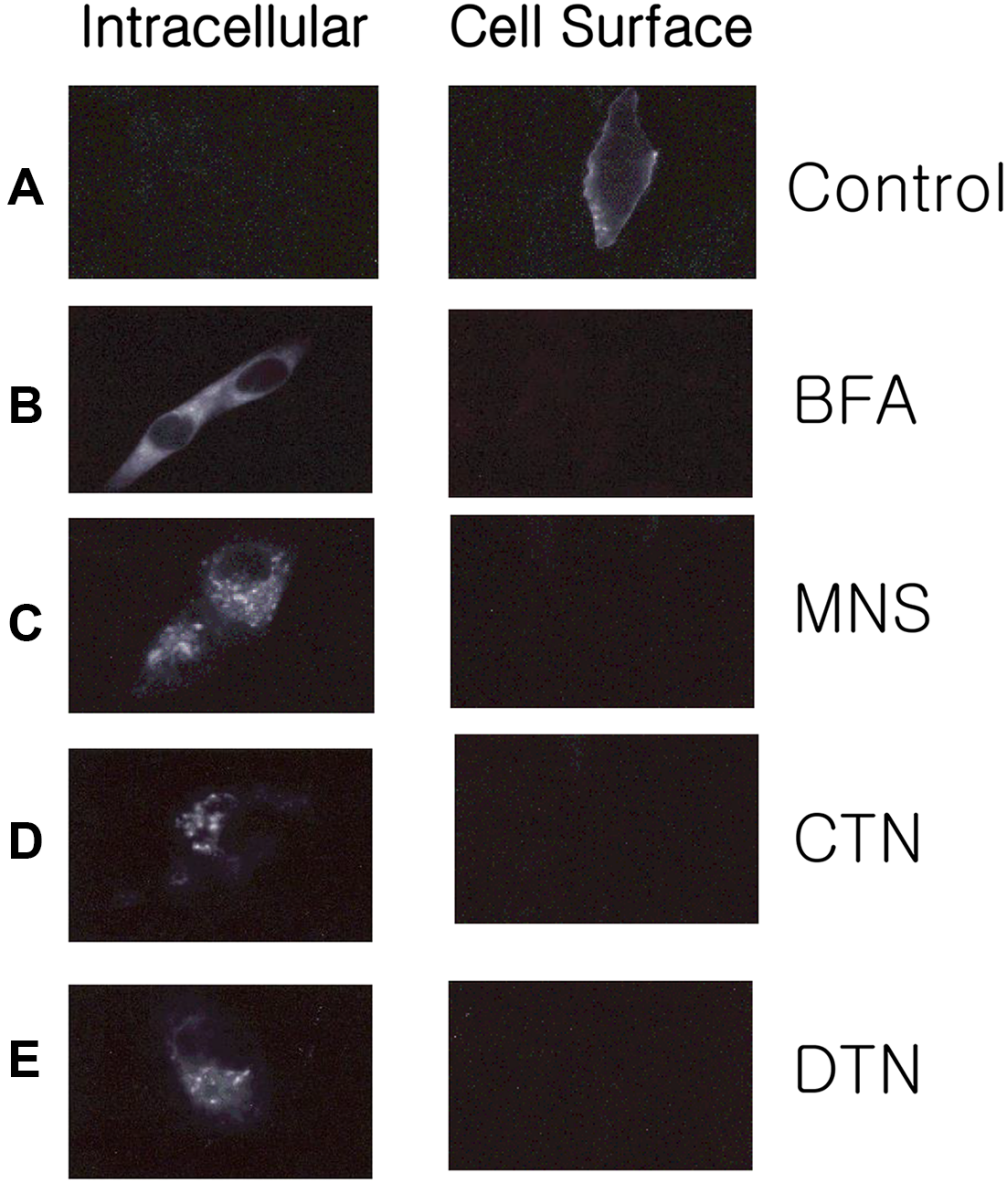
Effects of cryptotanshinone and dihydrotanshinone I on Golgi stack. BHK cells were put on a cover glass and infected with VSV, and the compounds (CTN and DTN; 10 μg/ml), BFA (1 μg/ml), or monensin (5 μg/ml) were added to the cultures at 1.5 h after infection. Dimethyl sulfoxide (DMSO) was added to the control cells. After 4.5 h of treatment with the drugs, cycloheximide (10 μg/ml) was added to each culture. The cultures were fixed after 1.5 h of incubation in the presence of cycloheximide and processed for immunofluorescence microscopy. For staining of intracellular VSV-G, the fixed cells were treated with –20°C methanol. Abbreviations: BHK, baby hamster kidney; VSV, vesicular stomatitis virus; CTN, cryptotanshinone; DTN, dihydrotanshinone I; BFA, Brefeldin A.

To investigate the intracellular accumulation of VSV-G, VSV-infected BHK cells were analyzed using immunofluorescence microscopy (Fig. 3). BFA and monensin inhibited the expression of VSV-G on the cell surface, resulting in its intracellular accumulation (Fig. 3B and 3C). We examined the localization of the VSV-G protein in CTN- and DTN-treated cells using indirect immunofluorescence (Fig. 3D and 3E). VSV-infected BHK cells were treated with CTN or DTN for 1 h, followed by treatment with cycloheximide for 1 h to inhibit protein synthesis. At 3 h post-infection, intracellular VSV-G protein was not detected in the control (Fig. 3A, intracellular), but was found in the CTN- and DTN-treated cells (Fig. 3D and 3E, intracellular). Strong intracellular staining was observed in CTN- and DTN-treated cells, indicating the suppression of cell surface expression and concomitant intracellular accumulation of VSV-G glycoproteins in the CTN- and DTN-treated cells.

The staining pattern reflects the localization of intracellular VSV-G accumulation. The localization of stained clouds in CTN- and DTN-treated cells was different from that in cells treated with BFA or monensin, which block intracellular trafficking from the endoplasmic reticulum to the Golgi or Golgi cisternae, respectively [22, 23]. The Golgi complex is fragmented by monensin, but remains localized to the perinuclear region [24]. In BFA-treated cells, Golgi components are redistributed to the endoplasmic reticulum or intermediate compartments [22-25]. In CTN- and DTN-treated cells, the clouds of immunofluorescence staining for VSV-G protein were not dispersed throughout the cytoplasm but were located at the Golgi stack. The immunostaining patterns of CTN- and DTN-treated cells were not similar to those of monensin- and BFA-treated cells (Fig. 3). The intact Golgi morphology in CTN- and DTN-treated cells suggests that these clouds of VSV-G are caused by disruption of the Golgi apparatus. However, further studies are required to determine the site(s) of the intracellular accumulation of VSV-G and their mechanism of inhibition.

### CTN and DTN Inhibit Intracellular Translocation of Envelope Glycoprotein of VSV

**Fig. 4:**
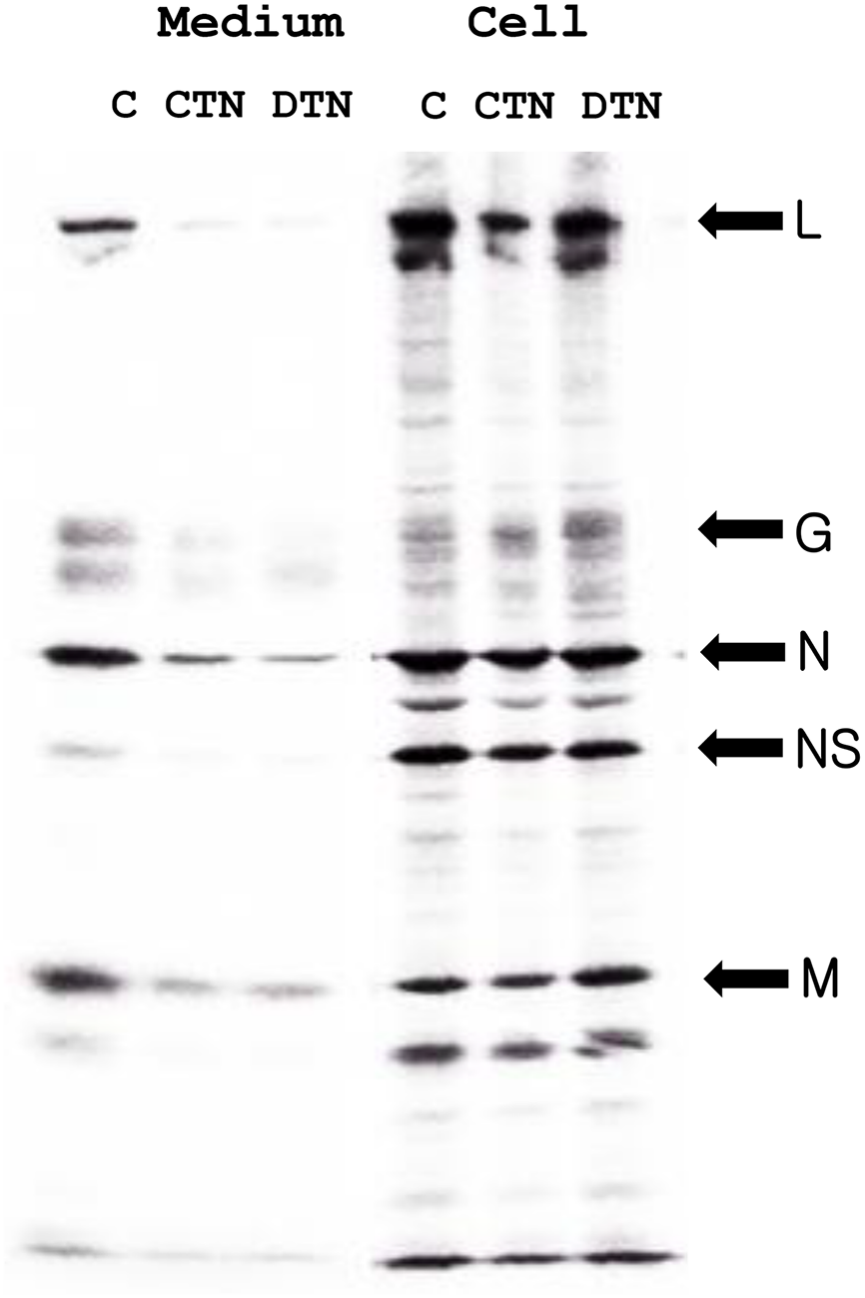
SDS-PAGE of [^35^S]-methionine-labeled products of VSV-infected cells. BHK cells were infected with VSV at an input multiplicity of ~10 PFU/cell. In the presence (lanes 2 and 5 for CTN; lanes 3 and 6 for DTN) or absence (lanes 1 and 4) of compounds (10 μg/ml), the cells were labeled with [^35^S]-methionine (0.37 MBq/ml) for 2 h and chased for 1 h in the presence of excess non-labeled methionine and cycloheximide. The cultures were divided into medium (lanes 1–3) and cell fractions (lanes 4–6), and analyzed using SDS-PAGE. Abbreviations: SDS-PAGE, SDS-polyacrylamide gel electrophoresis; VSV, vesicular stomatitis virus; BHK, baby hamster kidney; PFU, plaque forming units.

To elucidate the effects of CTN and DTN on the synthesis and intracellular translocation of the VSV G protein, VSV-encoded proteins were analyzed by SDS-PAGE. The synthesis of VSV-encoded proteins was not affected by CTN or DTN. In the control group, the amount of G protein on the cell surface was significantly higher than that in the intracellular space (Fig. 4). However, the release of VSV G protein into the medium fraction (Fig. 4, lanes 2 and 3) was greatly reduced in the presence of CTN and DTN (10 μg/ml). In contrast, G protein accumulated in the cell fraction (Fig. 4, lanes 5 and 6). Most nonstructural viral proteins were detected in the cell fraction but not in the medium fraction. This indicated that the release of G protein into the control medium fraction was not a consequence of the cytopathic effect of VSV replication. Extracellular proteinase K did not degrade the G protein accumulated in CTN- and DTN-treated cells. This result suggests that CTN and DTN block the cell surface expression of G proteins.

The SDS-PAGE band of the G protein in the cells in the presence of CTN and DTN was slightly faster than that of the mature protein secreted into the medium (Fig. 4, compare lane 1 and lanes 4–6). This suggested that the *N*-glycans of the G protein that accumulated in the presence of both compounds were immature. During intracellular trafficking to the cell surface, *N*-glycans undergo various modifications [26]. Thus, it was suggested that CTN and DTN block the intracellular translocation of glycoproteins before reaching the trans-Golgi network, where the processing of *N*-glycans is complete. In the presence of CTN and DTN (Fig. 4, lanes 5 and 6), the *N*-glycans of G protein accumulated intracellularly. Thus, cell surface expression of G-proteins was blocked in presence of CTN (20 μg/ml) and DTN (10 μg/ml).

### CTN and DTN Inhibit VSV-G Protein Trafficking by Fragmentation of Golgi

**Fig. 5:**
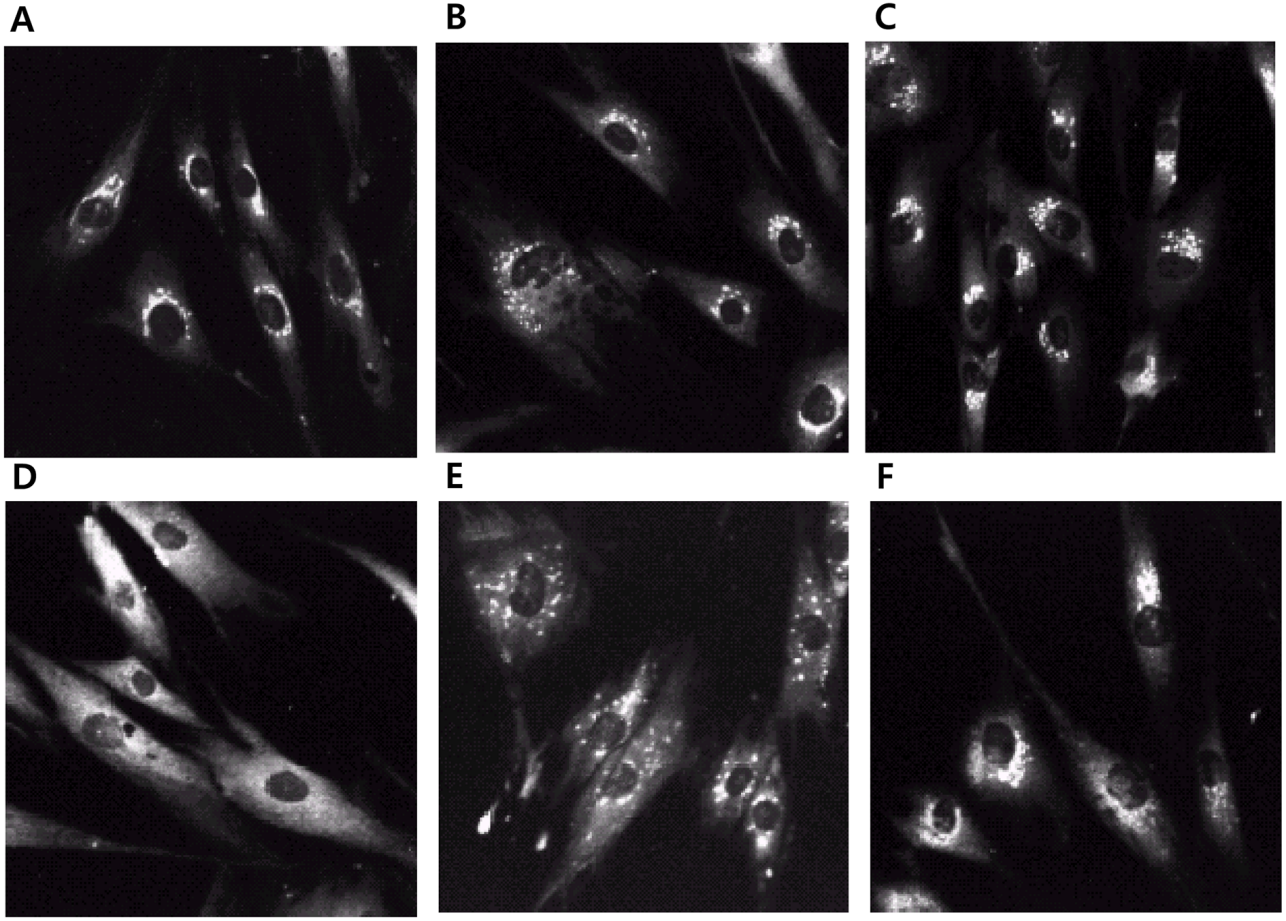
Fluorescence microscopy of Golgi stained with C6-NBD-ceramide. BHK cells were treated with CTN and DTN (10 μg/ml), BFA (1 μg/ml) or the vehicle at 37°C for 7 h. C6-NBD-ceramide was then added to each culture, and incorporated into the cells for 1 h. After 15 min of back-exchange, the cells were photographed without fixation. The compounds used are as follows; A, Control DMSO; B, CTN (10 μg/ml); C, DTN (10 μg/ml); D, BFA (1 μg/ml); E, Ilimaquinone (10 μg/ml); F, Monensin (10 μg/ml). Abbreviations: CTN, cryptotanshinone; DTN, dihydrotanshinone I; BFA, Brefeldin A.

To investigate the mechanism of action of CTN and DTN as glycoprotein trafficking inhibitors, we compared their effects with those of conventional inhibitors. As conventional glycoprotein inhibitors are involved in Golgi morphogenesis, we analyzed how CTN and DTN influence intracellular Golgi morphogenesis (Fig. 5). In the control group (Fig. 5A), the Golgi body remained close to the nucleus. However, Golgi fragmentation was observed following CTN and DTN treatment, but with a distinct pattern. Golgi fragments in the CTN- and DTN-treated cells (Fig. 5B and 5C) remained close to the nucleus but displayed unique patterns that differed from those in monensin treatment (Fig. 5F), which caused Golgi fragments to cluster near the nucleus, or BFA (Fig. 5D) and Ilimaquinone (Fig. 5E) treatment, which caused fragments to be widely distributed throughout the cytoplasm. In presence of BFA (Fig. 5D), the transportation of endoplasmic reticulum to the Golgi body is inhibited but the retrograde transport is not; therefore, the Golgi proteins moved to the endoplasmic reticulum, causing the Golgi body to disappear. This made the C6-NBD-ceramide show a color pattern similar to that of the endoplasmic reticulum. These findings suggest that the mechanisms of action of CTN and DTN as glycoprotein trafficking inhibitors differ from those of conventional inhibitors.

## Discussion

Glycosylation, which is the process of linking sugar moieties to proteins, is critical for diverse aspects of cell physiology, including cell attachment to the extracellular matrix and protein–ligand interactions within the cell. Glycoproteins are synthesized in the rough endoplasmic reticulum with concomitant glycosylation and are translocated intracellularly through the Golgi apparatus *en route* to their respective destinations, such as the cell surface, extracellular milieu, lysosomes, and other intracellular organelles [27]. This trafficking is accomplished by repeated budding and fusion of transport vesicles. Trafficking inhibitors are powerful tools for studying the mechanism of intracellular trafficking in mammalian cells [28]. However, only a few compounds have been shown to affect this intracellular trafficking. Trafficking inhibitors, such as leucinostatin A, can block the cell surface expression of viral glycoproteins without profoundly affecting their synthesis, which acts as a mitochondrial uncoupler leading to the inhibition of oxidative phosphorylation [29].

In our preliminary data, we observed that in most cases, a glycoprotein trafficking inhibitor produces free radicals in cells [14]. Based on these results, we hypothesized that radical-producing antibiotics could partially act as trafficking inhibitors of glycoproteins in cells. We screened CTN and DTN as radical-producing agents using a scavenging assay with *Bacillus subtilis*. To further verify whether CTN and DTN could inhibit the trafficking of glycoproteins in cells, we examined the effect of CTN and DTN on the blockade of cell surface expression of viral glycoproteins in BHK cells and changes in the Golgi complex and its cell morphology.

We first analyzed whether CTN and DTN inhibited SF without significantly affecting HN glycoprotein synthesis in NDV-infected BHK cells. Similar doses of CTN (20 μg/ml) and DTN (10 μg/ml) suppressed cytopathic effects and infectious virus production in VSV-infected BHK cells (Table 1 and Fig. 1). Blockade of the cell surface expression of NDV-HN and VSV-G glycoproteins by CTN and DTN was demonstrated, accompanied by intracellular accumulation of these viral glycoproteins (Fig. 3). This is the first report showing that CTN and DTN block the cell-surface expression of viral glycoproteins without profoundly affecting their synthesis. Immunofluorescence microscopy showed that the site(s) of intracellular accumulation of VSV G glycoprotein in CTN- and DTN-treated cells was different from that in BFA- and monensin-treated cells, suggesting that CTN and DTN may have a novel site of action in the glycoprotein trafficking pathway. The mechanism of protein trafficking inhibition by CTN and DTN remains to be elucidated, but we anticipate that CTN and DTN will be useful tools for investigating the mechanism of intracellular trafficking of glycoproteins.

Future research should aim to determine the specific step(s) at which the intracellular translocation of glycoproteins is inhibited. *N*-glycans transferred from the lipid intermediate in the rough endoplasmic reticulum to the polypeptide moiety undergo various modifications during trafficking from the Golgi apparatus to the cell surface. These modifications occur in defined compartments [30], and the blockage step(s) can be deduced by analyzing the structure of *N*-glycans.

In conclusion, we demonstrated that CTN and DTN can inhibit cell surface expression of VSV and suppress the trafficking of viral glycoproteins, particularly G-protein, to the Golgi apparatus. Further investigations focused on CTN and DTN can facilitate the development of novel antiviral drugs.

## Figures and Tables

**Table 1:** Effect of cryptotanshinone (CTN) and dihydrotanshinone I (DTN) on syncytium formation and glycoprotein synthesis in NDV-infected cells.

Parameter		Concentration (μg/ml)					
		1.3	2.5	5.0	10.0	20.0	40.0
CTN	α-glucosidase inhibition (%)	100	98.67 ± 2.31	80.00 ± 4.00	54.67 ± 4.62	36.00 ± 4.00	17.33 ± 2.31
	HAU (%)	100	100	100	98.67 ± 2.31	88.67 ± 7.02	54.00 ± 9.17
	SF	+	+	+	±	–	–
	CPE	–	–	–	–	+	+
DTN	α-glucosidase inhibition (%)	100	96.00 ± 4.00	70.67 ± 6.11	48.00 ± 4.00	29.33 ± 2.31	16.00 ± 0.00
	HAU (%)	100	100	88.67 ± 7.02	73.33 ± 6.11	56.67 ± 4.16	42.00 ± 4.00
	SF	+	+	+	–	–	–
	CPE	–	–	–	+	+	+

‘+’ indicates _____; ‘–’ indicates _______.

NDV, Newcastle disease virus; HAU, hemagglutination units; SF, syncytium formation; CPE, cytopathic effects.

## References

[1] Wattenberg BW (1990). Glycolipid and glycoprotein transport through the Golgi complex are similar biochemically and kinetically. Reconstitution of glycolipid transport in a cell free system. J. Cell Biol..

[2] Daniele S, David CG (2020). Direct trafficking pathways from the Golgi apparatus to the plasma membrane. Semin. Cell Dev. Biol..

[3] Muñiz M, Riezman H (2000). Intracellular transport of GPI-anchored proteins. EMBO J..

[4] Juan SB, Benjamin SG (2004). The mechanisms of vesicle budding and fusion. Cell.

[5] Aurora F, Massino M, Daniele DG, Alexandre AM, Galina VB (2013). Segregation of the Qb-SNAREs GS27 and GS28 into Golgi vesicles regulates intra-Golgi transport. Traffic.

[6] Heloise L, Han M, Woottum M, Bracq L, Bouchet J, Xie M (2020). Virus-mediated cell-cell fusion. Int. J. Mol. Sci..

[7] Muroi M, Suehara K, Wakusawa H, Suzuki K, Sato T, Nishimura T, Otake N (1996). Novel blockade of cell surface expression of virus glycoproteins by leucinostatin A. J. Antibiot..

[8] Liu HY, Yang P (2021). Small-molecule inhibition of viral fusion glycoproteins. Annu. Rev. Virol..

[9] Lad VJ, Gupta AK (2002). Inhibition of Japanese encephalitis virus maturation and transport in PS cells to cell surface by brefeldin A. Acta Virol..

[10] Thomas G (2002). Furin at the cutting edge: from protein traffic to embryogenesis and disease. Nat. Rev. Mol. Cell Biol..

[11] Sheetz MP, Pfister KK, Bulinski JC, Cotman CW (1998). Mechanisms of trafficking in axons and dendrites: implications for development and neurodegeneration. Prog. Neurobiol..

[12] Blondot ML, Bruss V, Kann M (2016). Intracellular transport and egress of hepatitis B virus. J. Hepatol..

[13] Wattenberg BW (1991). Analysis of protein transport through the Golgi in a reconstituted cell-free system. J. Electron Microsc. Tech..

[14] Lee DS, Lee SH, Noh JG, Hong SD (1999). Antibacterial activities of cryptotanshinone and dihydrotanshinone I from a medicinal herb, *Salvia miltiorrhiza* Bunge. Biosci. Biotech. Biochem..

[15] Lee DS, Lee SH (2000). Biological activity of dihydrotanshinone I: effects on Apoptosis. J. Biosci. Bioeng..

[16] Kim SL, Choi HS, Kim JH, Jeong DK, Kim KS, Lee DS (2019). Dihydrotanshinone-induced NOX5 activation inhibits breast cancer stem cell through the ROS/Stat3 signaling pathway. Oxid. Med. Cell. Longev..

[17] Lee DS, Kim SC, Kim DH, Kim JH, Park SP, Riu YC (2011). Screening of *Phellinus linteus*, a medicinal mushroom, for anti-viral activity. J. Korean Soc. Appl. Biol. Chem..

[18] Meihan L, Sascha CL, Jerry Y (2024). Reduction of hemagglutination induced by a SARS-CoV-2 spike protein fragment using an amyloid-binding benzothiazole amphiphile. Sci. Rep..

[19] Muroi M, Takasu A, Yamasaki M, Takatsuki A (1993). Folimycin (concanamycin A), an inhibitor of V-type H(+)-ATPase, blocks cellsurface expression of virus-envelope glycoproteins. Biochem. Biophys. Res. Commun..

[20] Kisch AL, Kelley RO, Eberle BJ (1972). Differential enhancement of R-type virus particles in polyoma-transformed BHK-21 cells by dimethyl sulfoxide. J. Natl. Cancer Inst..

[21] Laemmli UK (1970). Cleavage of structural proteins during the assembly of the head of bacteriophage T4. Nature.

[22] Jennifer LS, Lippincott-Schwartz J, Yuan L, Tipper C, Amherdt M, Orci L (1991). Brefeldin A's effects on endosomes, lysosomes, and the TGN suggest a general mechanism for regulating organelle structure and membrane traffic. Cell.

[23] Nakamura M, Kono Y, Takatsuki A (2003). Mepanipyrim, a novel inhibitor of pharmacologically induced Golgi dispersion. Biosci. Biotechnol. Biochem..

[24] Zizi M, Fisher RS, Grillo FG (1991). Formation of cation channels in planar lipid bilayers by brefeldin A. J. Biol. Chem..

[25] Sheung KL Yi C, Yu CT, Juan W, Angus HYL, Peter P (2009). BFA-induced compartments from the Golgi apparatus and *trans*-Golgi network/early endosome are distinct in plant cells. Plant J..

[26] Anna F, Angela KG, Sechi S, Giansanti MG (2020). The close relationship between the Golgi trafficking machinery and protein glycosylation. Cells.

[27] W Loscher, B Gericke (2020). Novel intrinsic mechanisms of active drug extrusion at the blood-brain barrier: potential targets for enhancing drug delivery to the brain?. Pharmaceutics.

[28] Fong F, Irwin MA (2012). Intracellular trafficking of P-glycoprotein. Int. J. Biochem. Cell Biol..

[29] M Muroi, K Suehara, H Wakusawa, K Suzuki, T Sato, T Nishimura (1996). Novel blockade of cell surface expression of virus glycoproteins by leucinostatin A. J. Antibiot (Tokyo).

[30] Kornfeld R, Kornfeld S (1985). Assembly of asparagine-linked oligosaccharides. Annu. Rev. Biochem..

